# Molecular Markers in Sex Hormone Pathway Genes Associated with the Efficacy of Androgen-Deprivation Therapy for Prostate Cancer

**DOI:** 10.1371/journal.pone.0054627

**Published:** 2013-01-24

**Authors:** Chia-Cheng Yu, Shu-Pin Huang, Yung-Chin Lee, Chao-Yuan Huang, Chia-Chu Liu, Tzyh-Chyuan Hour, Chun-Nung Huang, Bang-Jau You, Ta-Yuan Chang, Chun-Hsiung Huang, Bo-Ying Bao

**Affiliations:** 1 Division of Urology, Department of Surgery, Kaohsiung Veterans General Hospital, Kaohsiung, Taiwan; 2 Department of Pharmacy, Tajen University, Pingtung, Taiwan; 3 School of Medicine, National Yang-Ming University, Taipei, Taiwan; 4 Department of Urology, Kaohsiung Medical University Hospital, Kaohsiung, Taiwan; 5 Department of Urology, Faculty of Medicine, College of Medicine, Kaohsiung Medical University, Kaohsiung, Taiwan; 6 Cancer Center, Kaohsiung Medical University Hospital, Kaohsiung, Taiwan; 7 Department of Urology, National Taiwan University Hospital, College of Medicine National Taiwan University, Taipei, Taiwan; 8 Institute of Biochemistry, Kaohsiung Medical University, Kaohsiung, Taiwan; 9 Department of Chinese Medicine Resources, China Medical University, Taichung, Taiwan; 10 Department of Occupational Safety and Health, China Medical University, Taichung, Taiwan; 11 Department of Pharmacy, China Medical University, Taichung, Taiwan; 12 Sex Hormone Research Center, China Medical University Hospital, Taichung, Taiwan; Baylor College of Medicine, United States of America

## Abstract

Although most advanced prostate cancer patients respond to androgen-deprivation therapy (ADT), the efficacy is widely variable. We investigated whether the host genetic variations in sex hormone pathway genes are associated with the efficacy of ADT. A cohort of 645 patients with advanced prostate cancer treated with ADT was genotyped for 18 polymorphisms across 12 key genes involved in androgen and estrogen metabolism. We found that after adjusting for known risk factors in multivariate Cox regression models, *AKR1C3* rs12529 and *AR*-CAG repeat length remained significantly associated with prostate cancer-specific mortality (PCSM) after ADT (*P*≤0.041). Furthermore, individuals carrying two unfavorable genotypes at these loci presented a 13.7-fold increased risk of PCSM compared with individuals carrying zero (*P*<0.001). Our results identify two candidate molecular markers in key genes of androgen and estrogen pathways associated with PCSM after ADT, establishing the role of pharmacogenomics in this therapy.

## Introduction

Since the sex hormone signaling pathways play an important role in prostate cancer development, androgen-deprivation therapy (ADT) is still the standard systemic treatment for advanced prostate cancer. The majority of patients treated with ADT, which suppress androgen production or androgen receptor (AR) activity, show clinical improvement. Unfortunately, many patients relapse with a more aggressive form of prostate cancer termed castration-resistant prostate cancer (CRPC). Several mechanisms have been proposed for explaining the development of CRPC. The *AR* gene is amplified in about one third of cases [1]. Alteration of transcriptional coactivators and activation of signal pathways may enhance AR responses to low levels of androgens [2]. *AR* mutations in CRPC allow the receptor to be activated by weak androgens, other steroid hormones, or drugs [3]. In addition, direct measurements of intraprostatic androgens in castrated men with CRPC have shown that the levels are not significantly reduced compared with normal prostate, indicating that cancer cells generate significant active intracellular hormone levels to fuel their own growth [4].

Based on the above findings, genetic variants in genes of sex hormone metabolic pathways have been investigated as candidates for prostate cancer risk in many association studies [5], [6]. However, few studies have examined the association of these polymorphisms with prostate cancer progression and survival. Indeed, studies have shown the impact of variations in *CYP19A1*, *HSD3B1*, *HSD17B4*, *SLCO2B1*, and *SLCO1B3* on time to progression during ADT [7], [8], but there is still a lack of markers better defining lethal prostate cancer. In the present study, we sought to investigate the prognostic significance of common variants in sex hormone pathway genes on disease progression, prostate cancer-specific mortality (PCSM), and all-cause mortality (ACM) in a cohort of 645 prostate cancer patients receiving ADT.

## Patients and Methods

### Patient Recruitment and Data Collection

Six hundred and forty-five advanced prostate cancer patients were recruited between 1995 and 2009 from three medical centers in Taiwan: Kaohsiung Medical University Hospital, Kaohsiung Veterans General Hospital, and National Taiwan University Hospital, as previously described [9]–[14]. All patients were treated with ADT (orchiectomy or luteinizing hormone-releasing hormone agonist, with or without antiandrogen) and followed up prospectively to evaluate the efficacy of ADT. Data were collected on patients with disease baseline and clinicopathologic characteristics, as well as three treatment outcomes: time to progression, PCSM, and ACM. The prostate-specific antigen (PSA) nadir was defined as the lowest PSA value achieved during ADT treatment [15], [16]. Time to PSA nadir was defined as the duration of time it took for the PSA value to reach nadir after ADT initiation [17]. Disease progression was defined as a serial rise in PSA, at least two rises in PSA (>1 week apart), greater than the PSA nadir [7]. Initiation of secondary hormone treatment for rising PSA was also considered as a progression event. Time to progression was defined as the interval in months between the initiation of ADT and progression. In general, patients were followed every month with PSA tests at 3-monthly intervals. The cause of death was obtained by matching patients’ personal identification number with the official cause of death registry provided by the Department of Health, Executive Yuan, Taiwan. PCSM was defined as the interval from the initiation of ADT to death from prostate cancer. The ACM was defined as the period from the initiation of ADT to death from any cause. As the median PCSM and ACM had not been reached, the mean times to PCSM and ACM were estimated by Kaplan-Meier curves. This study was approved by the Institutional Review Board of Kaohsiung Medical University Hospital, Kaohsiung Veterans General Hospital, and National Taiwan University Hospital, and written informed consent was obtained from each participant.

### Selection of Single Nucleotide Polymorphisms (SNPs) and Genotyping

We selected 18 polymorphisms in 12 androgen and estrogen pathway genes with functional association with cancers according to the literature review. Genomic DNA was extracted from peripheral blood of patients and stored at −80°C until the time of study. Genotyping was performed by Sequenom iPLEX matrix-assisted laser desorption/ionization-time of flight mass spectrometry technology at the National Center for Genome Medicine, Academia Sinica, Taiwan. The average genotype call rate for these polymorphisms was 93.0% and each of the polymorphisms was in Hardy-Weinberg equilibrium (*P*>0.01). Ten percent of samples were blind duplicated for quality control and the genotype concordance was 100%.

### Statistical Analysis

Patient clinicopathologic characteristics were summarized as number and percentage of patients or median and interquartile range of values. The continuous factors were dichotomized at the median value within the cohort, with the exception of PSA nadir, which was dichotomized at 0.2 ng/mL because of its correlation with disease progression and PCSM [15], [18]. The associations of polymorphisms and clinicopathologic variables with time to progression, PCSM, and ACM were assessed using the Kaplan-Meier analysis with log-rank test. Since the function and the optimal genetic model for these polymorphisms remain unknown, a series of genetic models (based on the minor allele’s dominant: aa+Aa genotype versus AA genotype, recessive: aa genotype versus Aa+AA genotype, and additive: aa versus Aa versus AA) were tested. Multivariate analyses to determine the interdependency of polymorphisms and known prognostic factors, such as age at diagnosis, clinical stage, Gleason score, PSA at ADT initiation, PSA nadir, time to PSA nadir, and treatment modality, were carried out using Cox proportional hazards regression model. Higher order SNP-SNP interactions were evaluated using survival tree analysis by STREE software (http://c2s2.yale.edu/software/stree/), which uses recursive partitioning to identify subgroups of individuals with similar risk [19]. As we were testing 18 polymorphisms, false-discovery rates (*q* values) were calculated to determine the degree to which the tests for association were prone to false-positives [20]. *q* values were estimated using the R *q* value package. Statistical Package for the Social Sciences software version 16.0.1 (SPSS Inc., Chicago, IL) was used for other statistical analyses. A two-sided *P* value of ≤0.05 was considered statistically significant.

## Results

The study cohort consisted of 645 prostate cancer patients treated with ADT and the characteristics of patients were summarized in Table 1. The mean follow-up after ADT initiation in this cohort was 39 months (range, 3–125 months). Four hundred and forty-four patients had progressed with a median time to progression of 22 months. One hundred and sixty-two patients died, and 114 died of prostate cancer with the estimated mean times to ACM of 121 months and PCSM of 136 months. The clinical stage, Gleason score, PSA nadir, time to PSA nadir, and treatment modality were significantly associated with time to progression, PCSM, and ACM (*P*≤0.007). Age was only associated with ACM, and the PSA at ADT initiation was associated with PCSM and ACM.

**Table 1:** Clinicopathologic characteristics of the study population and analyses of factors that predicted disease progression, PCSM, and ACM during ADT.

Variable	No.* (%)	Disease progression			PCSM			ACM		
		No. of events*	Median (months)	*P* †	No. of events*	Estimated mean (months)	*P* †	No. of events*	Estimated mean (months)	*P* †
All patients	645	444	22		114	136		162	121	
Age at diagnosis, years										
Median (IQR)	73 (67–78)									
<73	306 (47.4)	215	21	0.368	49	136	0.154	57	131	**<0.001**
≥73	339 (52.6)	228	24		65	132		105	109	
Clinical stage at diagnosis										
T1/T2	192 (30.0)	120	25	**0.005**	12	147	**<0.001**	25	131	**<0.001**
T3/T4/N1	204 (31.8)	134	25		23	148		34	140	
M1	245 (38.2)	187	17		79	105		103	89	
Gleason score at diagnosis										
2–6	207 (32.8)	137	26	**0.004**	22	154	**<0.001**	37	140	**<0.001**
7	195 (30.9)	133	25		22	134		36	115	
8–10	230 (36.4)	164	17		69	104		87	92	
PSA at ADT initiation, ng/mL										
Median (IQR)	35.0 (11.3–130)									
<35	311 (49.9)	201	24	0.113	28	144	**<0.001**	49	131	**<0.001**
≥35	312 (50.1)	223	19		85	115		110	100	
PSA nadir, ng/mL										
Median (IQR)	0.19 (0.01–1.37)									
<0.2	320 (50.3)	198	31	**<0.001**	24	157	**<0.001**	41	144	**<0.001**
≥0.2	316 (49.7)	245	14		89	109		119	93	
Time to PSA nadir, months										
Median (IQR)	10 (5–17)									
<10	314 (49.4)	233	10	**<0.001**	71	121	**<0.001**	99	104	**<0.001**
≥10	322 (50.6)	210	32		42	146		61	134	
Treatment modality										
ADT as primary treatment	361 (56.2)	244	21	**0.007**	81	127	**<0.001**	116	111	**<0.001**
ADT for post RP/RT PSA failure	94 (14.6)	62	22		10	116		14	109	
Neoadjuvant/adjuvant ADT with RT	132 (20.6)	90	28		10	133		14	127	
Others	55 (8.6)	46	14		13	104		18	90	

Abbreviations: ADT, androgen-deprivation therapy; PCSM, prostate cancer-specific mortality; ACM, all-cause mortality; PSA, prostate-specific antigen; IQR, interquartile range; RP, radical prostatectomy; RT, radiotherapy.

* Column subtotals do not sum to 645 for no. of patients, 444 for no. of disease progression, 114 for PCSM, and 162 for ACM due to missing data.

† *P* values were calculated using the log-rank test.

*P*≤0.05 are in boldface.

A total of 18 polymorphisms in 12 genes involved in androgen and estrogen pathways were selected and genotyped (Table S1). One, 2, and 1 polymorphism achieved a *P* value of ≤0.05 for association with time to progression, PCSM, and ACM respectively, according to the univariate log-rank test. Median *AR*-CAG repeat length was 22 (interquartile range, 21–24), and there was an association with time to progression (*P* = 0.023, false-discovery rate *q* = 0.437) when analyzed as quartile groups (CAG repeat lengths <21, 21, 22–23, >23) (Table 2). To assess the impact of *AR*-CAG repeat length on disease progression beyond the clinical predictors, various known variables, including age at diagnosis, clinical stage at diagnosis, Gleason score at diagnosis, PSA at ADT initiation, PSA nadir, time to PSA nadir, and treatment modality, were evaluated together using Cox proportional hazards regression model. After adjustments for these predictors, the effect of *AR*-CAG repeat length on disease progression was attenuated.

**Table 2:** Genotyping frequencies and the association of genotype with disease progression during ADT.

Gene	Genotype	No. of patients	No. of events	Median (months)	*P* *	*q*	HR (95% CI)	*P* †
Polymorphism								
*AR*	<21	136	81	26	0.023	0.437	1.00	
CAG repeats	21	91	65	28			1.07 (0.76–1.51)	0.683
	22–23	165	111	23			0.92 (0.68–1.24)	0.589
	>23	198	149	19			1.11 (0.84–1.47)	0.472
	*P*-trend						1.02 (0.93–1.12)	0.620

Abbreviations: ADT, androgen-deprivation therapy; HR, hazard ratio; 95% CI, 95% confidence interval; PSA, prostate-specific antigen.

* *P* values were calculated using the log-rank test.

† HRs were adjusted for age, clinical stage, Gleason score, PSA at ADT initiation, PSA nadir, time to PSA nadir, and treatment modality.

*P*≤0.05 are in boldface.

**Figure 1:**
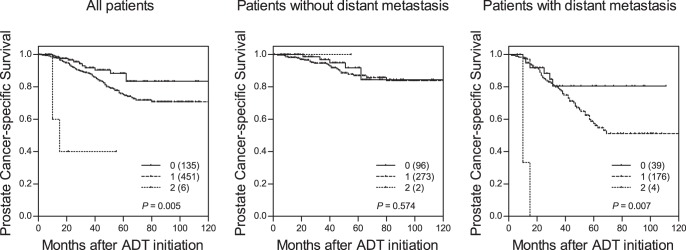
The influence of the genetic loci of interest on PCSM. Kaplan-Meier curves of time to PCSM during ADT for patients with 0, 1, or 2 unfavorable genotypes at the 2 genetic loci of interest in all patients (left), in patients without distant metastasis (middle), or in patients with distant metastasis (right). Numbers in parentheses indicate the number of patients.

*AKR1C3* rs12529 and *AR*-CAG repeat length were associated with PCSM (*P*≤0.029), and had a *q* value of 0.232 (Table 3). There was no association between these two polymorphisms and disease characteristics listed in Table 1 (data not shown). After adjusting for known variables, *AKR1C3* rs12529 and *AR*-CAG repeat length remained significant predictors for PCSM in patients receiving ADT (*P*≤0.041). A significant combined genotype effect on PCSM was also observed, and the hazard ratios (HRs) for PCSM increased as the number of unfavorable genotypes increased (HR 2.24, 95% confidence interval (CI) 1.20–4.18, *P* for trend = 0.011, Table 3 and Figure 1 left). Furthermore, individuals carrying 2 of these polymorphisms was associated with PCSM with a HR 13.7 (95% CI 3.60–52.4, *P*<0.001) compared with individuals carrying zero. Since metastatic disease typically has a poor prognosis, a substratification of high-risk patients based on the metastasis status at diagnosis was performed. The combined genotypes particularly had significant effects on PCSM in patients with distant metastasis (*P* for trend = 0.007; Figure 1 right), suggesting that these two polymorphisms might be independent predictors of clinical outcomes following ADT along with currently used prognostic factors in high-risk patients.

**Table 3:** Genotyping frequencies and the association of genotype with PCSM during ADT.

Gene	Genotype	No. of patients	No. of events	Estimated mean (months)	*P* *	*q*	HR (95% CI)	*P* †
Polymorphism								
***AKR1C3***	GG/GC	632	110	137	0.014	0.232	1.00	
** rs12529**	**CC**	8	3	43			**5.23 (1.60–17.1)**	**0.006**
***AR***	<21	137	12	143	0.029	0.232	1.00	
** CAG repeats**	21	91	18	131			1.62 (0.77–3.44)	0.206
	22–23	165	30	127			1.80 (0.91–3.56)	0.092
	**>23**	200	39	131			**2.02 (1.04–3.91)**	**0.037**
	*P*-trend						**1.22 (1.01–1.48)**	**0.041**
No. of unfavorable genotypes present‡								
	0	135	12	143	0.005		1.00	
	1	451	84	135			1.77 (0.95–3.27)	0.070
	**2**	6	3	29			**13.7 (3.60–52.4)**	**<0.001**
	*P*-trend						**2.24 (1.20–4.18)**	**0.011**

Abbreviations: ADT, androgen-deprivation therapy; HR, hazard ratio; 95% CI, 95% confidence interval; PSA, prostate-specific antigen.

* *P* values were calculated using the log-rank test.

† HRs were adjusted for age, clinical stage, Gleason score, PSA at ADT initiation, PSA nadir, time to PSA nadir, and treatment modality.

‡ Unfavorable genotypes refer to CC in AKR1C3 rs12529 and longer AR CAG lengths ≥21 repeats.

*P*≤0.05 are in boldface.

*CYP19A1* rs700519 was nominally associated with time to ACM in the univariate analysis (*P* = 0.050), and had a *q* value of 0.436 (Table 4). However, *CYP19A1* rs700519 did not reach significance after adjusting for known predictors in the multivariate analysis, possibly due to the correlations of *CYP19A1* rs700519 with clinical stage and time to PSA nadir (data not shown).

**Table 4:** Genotyping frequencies and the association of genotype with ACM during ADT.

Gene	Genotype	No. of patients	No. of events	Estimated mean (months)	*P* *	*q*	HR (95% CI)	*P* †
Polymorphism								
*CYP19A1*	CC/CT	622	154	122	0.050	0.436	1.00	
rs700519	TT	13	6	49			2.11 (0.92–4.82)	0.078

Abbreviations: ADT, androgen-deprivation therapy; HR, hazard ratio; 95% CI, 95% confidence interval; PSA, prostate-specific antigen.

* *P* values were calculated using the log-rank test.

† HRs were adjusted for age, clinical stage, Gleason score, PSA at ADT initiation, PSA nadir, time to PSA nadir, and treatment modality.

*P*≤0.05 are in boldface.

**Figure 2:**
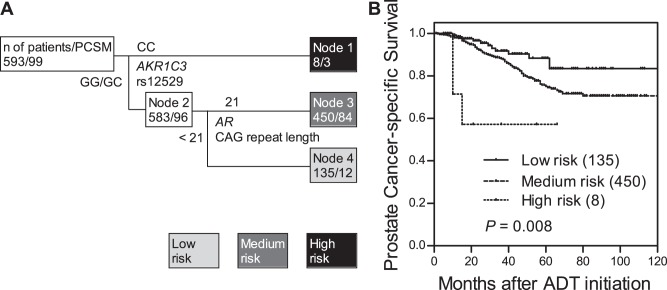
Potential higher order SNP-SNP interactions between *AKR1C3* rs12529 and *AR*-CAG repeat length. (A) Survival tree analysis identifies the interactions between *AKR1C3* rs12529 and *AR*-CAG repeat length. (B) Kaplan-Meier curves of time to PCSM during ADT based on the survival tree analysis. Numbers in parentheses indicate the number of patients.

We further used survival tree analysis to explored higher order SNP-SNP interactions among the SNPs that were associated with PCSM. The tree structure was first split by *AKR1C3* rs12529, following by *AR*-CAG repeat length, and resulted in 3 terminal nodes with low-, medium-, and high-risk for PCSM (Figure 2A). When using low risk node 4 as the reference group (GG/GC genotypes of *AKR1C3* rs12529 and *AR*-CAG repeat length <21), the HR was 1.77 (95% CI, 0.96–3.28, *P* = 0.069) for medium risk node 3, and 9.11 (95% CI, 2.47–33.6, *P* = 0.001) for high risk node 1. The time to PCSM decreased as the increase in risk classification (log-rank *P* = 0.008, Figure 2B). After adjusting for known variables, the genetic interaction profile between *AKR1C3* rs12529 and *AR*-CAG repeat length remained significant predictors for PCSM in patients receiving ADT (*P* for trend = 0.013).

## Discussion

We have identified two genetic polymorphisms, rs12529 in *AKR1C3* and CAG repeat in *AR*, retained their associations with PCSM after ADT while controlling for known prognostic factors, age at diagnosis, clinical stage, Gleason score, PSA level at ADT initiation, PSA nadir, and time to PSA nadir, suggesting that these host genetic factors add information above and beyond currently used predictors. Intriguingly, patients possessing a greater number of unfavorable alleles had a shorter survival following ADT.

A critical step in the synthesis of AR ligands involves the conversion of androstenedione to testosterone, which is catalyzed by 17β-hydroxysteroid dehydrogenases type 3 (HSD17B3) and type 5, also called aldo-keto reductase (AKR) 1C3. HSD17B3 is the predominant enzyme in catalyzing testosterone formation in testis, but synthesis of active androgens proceeds via AKR1C3 in prostate [21]. Several studies indicate that AKR1C3 is overexpressed in prostate cancer and its expression increases with the disease progression [22], [23]. AKR1C3 has also been suggested to contribute to the development of CRPC through the intratumoral formation of the active androgens [24]. Therefore, a specific inhibitor of AKR1C3 might have the potential to impact both hormone-sensitive prostate cancer and CRPC. Although the nonsynonymous polymorphism rs12529 causes a histidine to glutamine substitution at position 5 of AKR1C3, the amino acid is replaced by an amino acid of very similar chemical properties, leading to a conservative change. Nonetheless, rs12529 alters a putative exonic splicing enhancer motif that may cause alternative splicing regulatory effects, according to the prediction of FASTSNP [25]. Alternative splicing of AKR1C3 might regulate gene function and influence the efficacy of ADT. Moreover, *AKR1C3* rs12529 has also been associated with lung and bladder cancer risk [26], [27].

AR plays a pivotal role in prostate cancer development and progression. The factors that modify the function of AR might influence the progression of tumor to a castration-resistant state during ADT. The N-terminal transcriptional activation domain of the AR protein contains a CAG repeat, highly polymorphic in length, that affects the transactivation function of AR. Prior studies have shown an inverse relationship between CAG repeat length and AR transcriptional activation ability [28], and short CAG repeat lengths correlate with an increased risk of developing prostate cancer [29]. Although several studies have attempted to determine the role of *AR*-CAG repeat length on the outcomes of ADT, the results remain uncertain. Some studies showed that shorter CAG repeat length was correlated with better responses to hormonal therapy [30], [31], an observation consistent with the present study. On the other hand, other studies found that patients with better clinical responses to ADT had a longer CAG repeat length [32], [33], or in some cases, no correlation was found [34]–[37]. There are several possible explanations for the discrepancies in the literature. First, the measures of disease progression and the ethnic of study cohorts were different. It has been found that the prevalence of short CAG alleles was high in African-American men, intermediate in non-Hispanic whites, and low in Asians, suggesting racial differences in CAG repeat alleles. Two studies showing significantly improved responses to hormonal therapy for patients with shorter CAG repeat lengths were in Asians, Japanese [31] and Chinese (this study). Second, the contraction of CAG repeat lengths occur frequently within prostate tumors, and the lengths differ from those found in the germline samples [38]. The present and several previous studies evaluated germline *AR*-CAG repeat lengths in peripheral blood samples, but the actual repeat lengths within the prostate tumors might play a more critical role in response to ADT. Finally, AR has recently been suggested to function as a tumor suppressor in epithelium to suppress prostate tumor invasion and metastasis [39]. Also, several reports have shown that higher AR expression and pretreatment testosterone levels predict better response to endocrine therapy [40]–[42]. Consequently, combined with our results, higher transactivated AR with shorter CAG repeats might inhibit prostate cancer metastasis and predict a good prognosis on ADT. The goal of ADT is to inhibit AR and prevent androgens from reaching prostate cancer cells, but the development of CRPC almost always occurs. Several mechanisms have been proposed to explain the development of CRPC including *AR* amplifications, alteration of its coregulators rendering AR signaling sensitive to low concentrations of androgen, and *AR* mutations allowing the receptor to be reactivated by other steroids as well as by antiandrogens. Therefore, other factors that might influence the activity of AR, such as AR coregulators and *AR* mutations, should also be studied in conjunction of *AR*-CAG repeats to allow a more comprehensive analysis.

In conclusion, most prostate cancer patients will have an indolent form of disease, but aggressive prostate cancer is still the second leading cause of cancer deaths in men of the United States. New biomarkers to help distinguish between lethal and indolent prostate cancer are urgently needed. Of the 18 polymorphisms in the 12 sex hormone pathway genes, we identified two polymorphisms in *AKR1C3* and *AR* that were associated with PCSM. Our cohort consisted of only Chinese Han population, and the results reported here are limited by multiple comparisons. Further work is necessary to characterize these polymorphisms and determine how to ultimately translate these findings into clinical practice.

## Supporting Information

Table S1 Genotyped polymorphisms and the *P* values of their association with time to progression, PCSM, ACM during ADT.
(DOC)
