# Supplementary material for: Molecular Markers in Sex Hormone Pathway Genes Associated with the Efficacy of Androgen-Deprivation Therapy for Prostate Cancer
Source: PLoS One. 2013 Jan 24;8(1):e54627. doi: 10.1371/journal.pone.0054627 (PMC3554749; doi:10.1371/journal.pone.0054627)
Supplement: Table S1 — Genotyped polymorphisms and the P values of their association with time to progression, PCSM, ACM during ADT. (DOC) [file pone.0054627.s001.doc]

**Table S1. Genotyped polymorphisms and the *P* values of their association with time to progression, PCSM, ACM during ADT**

| Gene | polymorphism | Log-rank *P* | | | | | | | | |
| --- | --- | --- | --- | --- | --- | --- | --- | --- | --- | --- |
|  |  | Progression | | | PCSM | | | ACM | | |
|  |  | Additive | Dominant | Recessive | Additive | Dominant | Recessive | Additive | Dominant | Recessive |
| ***AKR1C3*** | **rs12529** | 0.870 | 0.683 | 0.202 | 0.542 | 0.876 | **0.014** | 0.511 | 0.731 | 0.077 |
| *AKR1C3* | rs7741 | 0.912 | 0.912 | - | 0.433 | 0.433 | - | 0.904 | 0.904 | - |
| ***AR*** | **CAG repeats** | **0.023** | - | - | **0.029** | - | - | 0.299 | - | - |
| *COMT* | rs4680 | 0.287 | 0.394 | 0.333 | 0.168 | 0.131 | 0.590 | 0.544 | 0.579 | 0.667 |
| *CYP17A1* | rs743572 | 0.859 | 0.778 | 0.531 | 0.613 | 0.713 | 0.643 | 0.470 | 0.494 | 0.632 |
| *CYP19A1* | rs10046 | 0.496 | 0.098 | 0.589 | 0.444 | 0.393 | 0.701 | 0.645 | 0.819 | 0.597 |
| ***CYP19A1*** | **rs700519** | 0.951 | 0.898 | 0.837 | 0.227 | 0.365 | 0.150 | 0.157 | 0.329 | **0.050** |
| *CYP1B1* | rs1056836 | 0.871 | 0.854 | 0.995 | 0.640 | 0.699 | 0.659 | 0.757 | 0.706 | 0.918 |
| *CYP3A4* | rs2740574 | 0.335 | 0.223 | 0.670 | 0.439 | 0.421 | 0.512 | 0.363 | 0.344 | 0.439 |
| *ESR1* | rs2234693 | 0.816 | 0.442 | 0.588 | 0.903 | 0.316 | 0.304 | 0.300 | 0.121 | 0.977 |
| *ESR1* | rs9340799 | 0.882 | 0.953 | 0.627 | 0.077 | 0.080 | 0.375 | 0.221 | 0.106 | 0.806 |
| *HSD17B4* | rs7737181 | 0.639 | 0.796 | 0.257 | 0.245 | 0.289 | 0.422 | 0.234 | 0.290 | 0.341 |
| *HSD3B1* | rs1856888 | 0.519 | 0.519 | - | 0.627 | 0.627 | - | 0.181 | 0.181 | - |
| *KLK3* | rs266882 | 0.866 | 0.526 | 0.220 | 0.140 | 0.124 | 0.749 | 0.089 | 0.087 | 0.584 |
| *KLK3* | rs4802754 | 0.598 | 0.906 | 0.376 | 0.667 | 0.898 | 0.501 | 0.449 | 0.480 | 0.615 |
| *KLK3* | rs925013 | 0.666 | 0.869 | 0.529 | 0.447 | 0.615 | 0.422 | 0.299 | 0.381 | 0.411 |
| *SRD5A2* | rs523349 | 0.400 | 0.328 | 0.771 | 0.410 | 0.898 | 0.180 | 0.954 | 0.385 | 0.318 |
| *SRD5A2* | rs676033 | 0.720 | 0.576 | 0.992 | 0.296 | 0.566 | 0.258 | 0.377 | 0.643 | 0.328 |

Abbreviations: ADT, androgen-deprivation therapy; PCSM, prostate cancer-specific mortality; ACM, all-cause mortality.

*P* ≤ 0.05 are in boldface.
